# First-episode Psychosis and Migration in Italy: Results from a Study in the Italian Mental Health Services (Pep-Ita Study)

**DOI:** 10.1007/s10903-021-01168-w

**Published:** 2021-03-10

**Authors:** Ilaria Tarricone, Giuseppe D’Andrea, Viviana Storbini, Mauro Braca, Silvia Ferrari, Corinna Reggianini, Marco Rigatelli, Carla Gramaglia, Patrizia Zeppegno, Eleonora Gambaro, Mario Luciano, Alessio Ceregato, Mario Altamura, Giuseppe Barrasso, Diego Primavera, Bernardo Carpiniello, Orlando Todarello, Vanna Berlincioni, Francesca Podavini, Craig Morgan, Robin M. Murray, Marta Di Forti, Roberto Muratori, Domenico Berardi

**Affiliations:** 1 Department of Medical and Surgical Sciences, Bologna Transcultural Psychosomatic Team (BoTPT), University of Bologna, Viale C. Pepoli 5, 40123 Bologna, Italy; 2 Department of Mental Health and Pathological Addiction, Local Health Authority, Bologna, Italy; 3 Department of Biomedical and NeuroMotor Sciences, Psychiatry Unit, University of Bologna, Bologna, Italy; 4 Department of Biomedical, Metabolic and Neural Sciences, University of Modena & Reggio Emilia, Modena, Italy; 5 Department of Translational Medicine, Institute of Psychiatry, Università del Piemonte Orientale, Novara, Italy; 6 Department of Psychiatry, University of Campania Luigi Vanvitelli, Naples, Italy; 7 Mental Health Department A.S.L. TO4, Community Mental Health Service, Chivasso, TO Italy; 8 Department of Clinical and Experimental Medicine, Section of Psychiatry and Clinical Psychology, University of Foggia, Foggia, Italy; 9 Andria Community Mental Health Centre, Andria, BAT Italy; 10 Department of Medical Science and Public Health-Section of Psychiatry, University of Cagliari, Cagliari, Italy; 11 U.O. di Psichiatria – Azienda Ospedaliero-Universitaria “Consorziale Policlinico” Bari; Dipartimento Di Scienze Mediche Di Base, Neuroscienze Ed Organi Di Senso, Università Degli Studi Aldo Moro Di Bari, Bari BA, Italy; 12 Department of Brain and Behavioral Sciences, University of Pavia, Pavia, Italy; 13 Institute of Psychiatry, Psychology & Neuroscience, King’s College London, 16 De Crespigny Park, London, SE5 8AF UK

**Keywords:** First-episode psychosis, Migrants, Pathway to care

## Abstract

*Background*: Migrants present high rates of psychosis. A better understanding of this phenomenon is needed. *Methods*: We conducted a multicentre First-Episode Psychosis (FEP) prospective study over two years (January 2012–December 2013) to evaluate first-generation migrants presenting with FEP at the participating Community Mental Health Centers (CMHCs). *Results*: 109 FEP migrants were identified. Almost half of them were highly educated, employed and in a stable affective relationship. The average age was 32.8 (± 9.8) years, and the average length of stay in Italy was 8.6 (± 8.8) years. About 2/3 of patients were referred to CMHCs following Emergency Department access or psychiatric admission. Conclusions: Our finding of a “high functioning portrait” of FEP migrants allow us to hypothesize that a high burden of negative psychosocial factors is likely to be needed for the FEP onset. Furtherly, mental health services should implement more appropriate resources and organizational methods to respond to migrants’ health needs.

## Introduction

Nowadays, the mental health of migrants is a major individual and public health issue in the EU. Unlike other European countries such as England and France, where migration has been a structural component of society for centuries, Italy is only recently facing a rapid transformation of identity, changing from a country of emigration to a country of immigration. From the beginning of 2012 to the end of 2013, the number of migrants in Italy increased from 4.052.081 to 4.922.085 (+ 21.5%).

Epidemiological evidence showed an increased incidence of psychotic disorders among several migrant populations compared to natives [1–6]. To explain the high rates of psychosis in migrants, Morgan et al. in 2010 [5] proposed a socio-developmental pathway to psychosis, highlighting the putative causal role of adverse social conditions and experiences. Throughout the whole migration process (from the decision to migrate to the adjustment to the new society), migrants may be exposed to several social disadvantages, including, among others, unemployment, poor living conditions, social isolation, and discrimination. To our knowledge few studies have investigated the risk factors for the onset of psychotic disorders in first-generation migrants in Italy [6–8].

Despite the high rate of psychotic disorders in migrants, access to mental health care for migrants can be particularly difficult [9–11]. In Italy, health care coverage is unlimited and free of charge for the whole population. Psychiatric care is delivered by general hospital psychiatric wards for acute admissions, and Community Mental Health Centres (CMHCs) providing psychiatric care to geographically defined areas. Non-resident people, such as migrants, can access care as much as the resident population for urgent and/or necessary cases, and are referred to the same CMHCs as the general population [12, 13]. The Italian National Health Service (NHS) organization would facilitate access to care for migrants presenting FEP in Italy. The health services organization showed important differences among Italian regions [14]. Previous studies showed that migrants with mental disorders might follow different Pathways to Care (PtC) in Italy [10, 11].

## Objectives

The present study aims to:

1. describe the socio-demographic and clinical features of migrants with FEP consecutively recruited in 9 Community Mental Health Centers (CMHCs) in Italy;
2. explore the clinical characteristics and PtC of migrant patients at the onset of psychosis and evaluate possible differences between centres.

This study is part of the Italian multicentre research project PEP-Ita (First Episode Psychosis – Italy), a prospective study conducted over a two-year period (1st January 2012–31th December 2013) to evaluate first generation migrants with psychotic onset presenting for the first time to the 9 participating CMHCs [15].

## Methods

### Study Design

The aims and methods of the PEP-Ita project have already been described in a previous work [16]. The design of the PEP-Ita study was drawn in accordance with the EUGEI project (gene x environment interaction European study) (No. HEALTH-F2-2009-241909) [17].

The centres participating in the study collected relevant data on all new cases of migrants seeking treatment for FEP for the first time during the recruitment period (1^st^ January 2012-31^th^ December 2013).

Inclusion criteria were:

1. age between 18 and 64 years;
2. diagnosis of psychotic episode, defined accordingly to the diagnostic criteria of DSM-IV-TR [18] by the presence of at least one of the following symptoms: (a) delusions, (b) hallucinations, (c) disorganized speech, (d) disorganized behaviour;
3. residence in the study catchment area of the centres involved.
4. first psychiatric contact

Exclusion criteria were:

1. diagnosis of moderate or severe mental retardation, according to the criteria of the DSM-IV-TR and confirmed by the administration of the WAIS-III-abbreviated version [19];
2. general medical conditions that do not allow a reliable clinical evaluation of the patient;
3. history of previous psychotic episodes treated with adequate antipsychotic therapy.

### Setting and Study Population

The study catchment areas were defined in terms of the Census Area covered by participating CMHCs. Based on data from the Italian National Institute of Statistics (ISTAT) [20], the whole catchment area was of 2.135.145 inhabitants and 6.5% of migrants. Considering a conservative estimate of yearly incidence cases of 40/100.000 among migrants [6], we expected to recruit 111 FEP migrants cases by year 2. We conducted a leakage study to identify any subject that may have been missed during the critical data collection period. To do so, we reviewed all new mental health service registration forms and interrogated the computerized information systems.

**Fig. 1:**
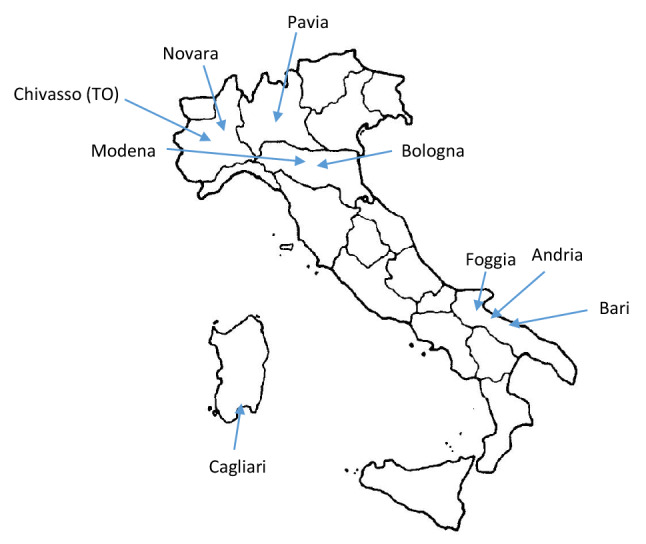
Distribution of study centres

The 9 participating centres, located in different regions of the national territory, were: Andria, Bari, Bologna, Cagliari, Chivasso, Foggia, Modena, Novara and Pavia (see Fig. 1 and Table 1).

**Table 1:** Description of study centres

Study centres	Mental Health Services and Universities	Population (n)	Density (n/km^2^)	Migrants (n; %)
Andria (BAT)	Andria Community Mental Health Centre	100,052	248,34	1434 (1.4)
Bari (BAR)	U.O. di Psichiatria—Azienda Ospedaliero Universitaria “Consorziale Policlinico” Bari	315,933	2691,32	6705 (2.1)
Bologna (BOL)	Bologna Transcultural Psychiatric Team (BoTPT)—Department of Medical and Surgical Sciences, University of Bologna Department of Mental Health and Pathological Addictions, Local Health Trust, Bologna	371,337	2636,24	44,023 (11.9)
Cagliari (CAG)	Clinica Psichiatrica—Università degli Studi di Cagliari	149,883	1763,04	4542 (3.0)
Chivasso (CHV)	Department of Mental Health A.S.L. TO4	25,914	505,75	1768 (6.8)
Foggia (FOG)	Department of Clinical and Experimental Sciences, Section of Psychiatry and Clinical Psychology, University of Foggia	147,036	288,72	2694 (1.8)
Modena (MOD)	U.O. Psichiatria Ospedaliero-Universitaria Modena Centro, University of Modena and Reggio Emilia	179,149	977,92	24,166 (13.5)
Novara (NOV)	SC Psichiatria, AOU Ospedale Maggiore della Carità, Dipartimento di Medicina Traslazionale, Università del Piemonte Orientale Amedeo Avogadro, Novara, Italy	101,952	989,34	11,776 (11.6)
Pavia (PAV)	Department of Brain and Behavioral Sciences, University of Pavia	68,280	1079,62	5648 (8.3)

The research was coordinated by the Bologna Transcultural Psychosomatic Team (BoTPT) of the University of Bologna [21]. The BoTPT, in collaboration with the Department of Mental Health of the Bologna Local Health Authority, facilitates psychosocial interventions for migrant populations and examines the key issues concerning the mental health of migrants.

## Assessment

For each patient we collected the following information: (1) socio-demographic data (gender, age, date of birth, country of origin, ethnicity, length of stay in Italy, marital status, level of education, employment status, housing status); (2) medical and psychiatric history of patients and their families; (3) clinical data (psychiatric diagnoses, medical comorbidities, age at onset and at first contact with CMHC, duration of untreated psychosis (DUP), previous psychiatric admissions); (4) PtC: referral by general practitioners, by ER or general hospitals, by psychiatric ward, by other psychiatric services, by the police or by judicial authorities, informal referral (by relatives OR self-referral).

Those characteristics were derived from clinical and electronic charts in the CMHCs involved.

Clinical diagnoses were made by psychiatrists in each centre according to DSM-IV-TR criteria [18] and were subsequently grouped into four diagnostic clusters: non-affective psychosis – schizophrenia (F20.00-F20.59), schizophreniform disorder (F20.8), schizoaffective disorder (F25.0-F25.1), delusional disorder (F22.0); affective psychosis—major depressive disorder with psychotic symptoms (F32.3 and F33.3), bipolar disorder with psychotic symptoms (F30.2, F31.2 and F31.5), mood disorder NOS with psychotic symptoms (F39); NOS psychosis (F29); other diagnoses—brief psychotic disorder (F23.80-F23.81), substance-induced psychotic disorders (F10.51, F10.52, F11.51, F11.52, F12.51, F12.52, F13.51, F13.52, F14.51, F14.52, F15.51, F15.52, F16.51, F16.52, F18.51, F18.52, F19.51 and F19.52).

## Statistical Analysis

Data were analyzed using SPSS for Windows Version 14.0. Methods for inter-group comparison (*p* = 0.05) included χ^**2**^ test for category-dependent variables (the Bonferroni correction was applied where required), independent t-test analysis (one-way analysis of variance) and analysis of covariance for continuous dependent variables.

The study protocol has been approved by ethics committee of the coordinating Centre (Bologna, protocol n. 113/2006/U) and has been submitted to each local ethics committee. The study was conducted in accordance with the Declaration of Helsinki.

## Results

### Socio-demographic Characteristics

109 FEP migrants were identified during the study period. Table 2 shows socio-demographic features of included migrant patients. 36% of the sample was from Eastern Europe, 27% from Asia, 16% from Sub-Saharan Africa, 15% from Maghreb, 6% from Central and South America and only 1% from Western Countries. The average age at the time of evaluation was 32.8 (± 9.8) years, while the average age at onset was 31.8 (± 9.9) years. The average length of stay in Italy was 8.6 (± 8.8) years. Almost half of the sample was single, while the other half was currently or had been in a stable affective relationship (married, cohabitant, in a stable relationship, divorced or widower). More than half had a high school diploma or higher degree. About 40% were employed, while one third of patients were unemployed and another 22% were economically inactive (9, 8% housewives, 12, 11% retired, 1, 0.9% invalids). One third of patients lived with their acquired family (partner/spouse and/or children), while 27% lived with their family of origin (mother, father, siblings, etc.) and 21% lived alone.

**Table 2:** Description of the sample – sociodemographic features

Gender	
Men	60 (55%)
Women	49 (45%)
Region of origin	
Maghreb	16 (15%)
Sub-Saharan Africa	17 (16%)
Asia	29 (27%)
Eastern Europe	39 (36%)
CS America	7 (6%)
Western Countries	1 (1%)
Age at first contact	32.8 ± 9.8
Age at onset^1^	31.8 ± 9.9
Lenght of stay in Italy^2^	8.6 ± 8.8
Marital status	
Single	53 (49%)
Married/cohabiting	46 (42%)
Stable relationship	4 (4%)
Divorced/separated	5 (5%)
Widower	1 (1%)
Title of study^3^	
Illitterate/Primary school	2 (2%)
Middle school	35 (43%)
High school	31 (38%)
University degree/higher	13 (16%)
Employment status^4^	
Unemployed	22 (34%)
Economically inactive	14 (22%)
Student	3 (5%)
Part-time job	10 (16%)
Full-time job	15 (23%)
Housing status^5^	
Alone	23 (21%)
With family of origin	29 (27%)
With own family	36 (34%)
With friends	4 (4%)
Other	15 (14%)
Total	109 (100%)

^1^2 missing

^2^51 missing

^3^28 missing

^4^45 missing

^5^2 missing

Table 3 shows socio-demographic characteristics of patients by study centres. More than half of the sample (62%) lived in Northern Italy (Bologna, Modena, Novara, Pavia, Chivasso), while 39% lived in Southern Italy or on the islands (Bari, Foggia, Andria, Cagliari). Among study centres we found significant differences in relation to regions of origin of the patients (*p* = 0.002). The most represented region of origin was Asia in Bologna (36%) as well as in Bari (53%), while in Pavia, Andria and Foggia most of migrants came from Eastern Europe (60%, 50% and 44% respectively), in Modena from Sub-Saharan Africa (33%) and in Novara from Maghreb (36%). Marital status showed a trend of statistical differences, with more single people in Bologna (45%), Bari (65%), Modena (58%) and Pavia (60%) and more married people in Andria (62%). A trend for a significantly higher number of migrants with a high school diploma has been found in Bologna, Andria, Chivasso and Novara. In Bologna and Modena most patients lived with their family of origin (41% and 43% respectively); in Andria (50%) and Novara (50%) with acquired family; alone in Bari (35%) and Foggia (40%).

**Table 3:** Socio-demographic characteristics of the sample by study centre

	BOL	BAT	CAG	CHV	BAR	FOG	MOD	NOV	PAV	*p*
Gender Man	10 (45%)	4 (50%)	-	1 (50%)	12 (71%)	10 (62%)	14 (58%)	5 (36%)	4 (80%)	0.469
Age Current age Age at onset^1^	32.7 ± 11.7 32.3 ± 11.6	31.1 ± 7.7 28.2 ± 11	36 35	36.5 ± 14.8 ?	30.6 ± 8.6 29.8 ± 9.3	30.5 ± 10.6 30.3 ± 10.8	32.6 ± 8.7 32.5 ± 8.6	39.2 ± 9.1 36.7 ± 9.1	32.6 ± 9.1 31.8 ± 9.7	0.260 0.496
Region of origin Maghreb S–S Africa Asia Eastern Europe CS America W Countries	5 (23%) 1 (4%) 8 (36%) 6 (27%) 2 (9%) -	2 (25%) 1 (12%) 1 (12%) 4 (50%) - -	- - - 1 (100%) - -	- - - 2 (100%) - -	- 3 (18%) 9 (53%) 5 (29%) - -	1 (6%) 3 (19%) 4 (25%) 7 (44%) 1 (6%) -	3 (12%) 8 (33%) 6 (25%) 7 (29%) - -	5 (36%) - 1 (7%) 4 (29%) 4 (29%) -	- 1 (20%) - 3 (60%) - 1 (20%)	0.002
Marital Single Married Divorced Widower Stable relationship	10 (45%) 9 (41%) - - 3 (14%)	3 (37%) 5 (62%) - - -	- 1 (100%) - - -	1 (50%) - 1 (50%) - -	11 (65%) 6 (35%) - - -	8 (50%) 8 (50%) - - -	14 (58%) 8 (33%) 1 (4%) 1 (4%) -	3 (21%) 7 (50%) 3 (21%) - 1 (7%)	3 (60%) 2 (40%) - - -	0.125
Employment status^2^ Unemployed	8 (36%)	?	1 (100%)	1 (100%)	1 (25%)	2 (67%)	3 (21%)	5 (36%)	1 (20%)	0.571
Title of study^3^ High school/higher	13 (62%)	4 (100%)	-	2 (100%)	6 (46%)	4 (44%)	6 (46%)	8 (57%)	1 (25%)	0.145
Housing status^4^ Alone With family of origin With own family With friends Other	2 (9%) 9 (41%) 6 (27%) 3 (14%) 2 (9%)	1 (12%) - 4 (50%) - 3 (37%)	- - 1 (100%) - -	- 1 (50%) 1 (50%) - -	6 (35%) 3 (18%) 3 (18%) - 5 (29%)	6 (40%) 3 (20%) 5 (33%) 1 (7%) -	3 (13%) 10 (43%) 7 (30%) - 3 (13%)	4 (29%) 1 (7%) 7 (50%) - 2 (14%)	1 (20%) 2 (40%) 2 (40%) - -	0.141
Total	22 (20%)	8 (7%)	1 (1%)	2 (2%)	17 (16%)	16 (15%)	24 (22%)	14 (13%)	5 (5%)	

*BOL* Bologna, *BAT* Andria, *CAG* Cagliari, *CHV* Chivasso (TO), *BAR* Bari, *FOG* Foggia, *MOD* Modena, *NOV* Novara, *PAV* Pavia

^1^2 missings

^2^45 missings

^3^28 missings

^4^2 missings

? = 100% missings

### Clinical Characteristics

Almost 2/3 of patients were referred by hospitals (psychiatric wards, Emergency Rooms, other hospital wards), 12% from other psychiatric services (private specialists or private clinics), 11% made an informal access (auto-referral or being referred by relatives), 8% were referred by general practitioners and 5% by police or judicial authorities. The average duration of untreated psychosis (DUP) was 9.3 weeks (± 21.6). The most frequent diagnosis was non-affective psychosis (42%), with schizophrenia representing 9% of the total sample, followed by psychosis NOS (33%) and affective psychoses (21%); the remaining 5% had other diagnoses (brief psychotic disorder, substance-induced psychotic disorders). One patient in six (35% of patients for whom the information was available) presented medical comorbidities: 8% of patients were suffering from internal diseases (anaemia, gastroesophageal reflux disease, high blood pressure), 3% from metabolic diseases (diabetes, hypercholesterolemia), 2% from traumatic diseases (bone fractures, other injuries) and 3% from other diseases (autoimmune, neoplastic diseases). 21 (19.3%) of the patients use substances with cannabis being by far the most used (by 20 out of the 21 substance users) (Table 4).

**Table 4:** Description of the sample – clinical features

Referral^1^	
GP	8 (8%)
ER/General hospital	39 (39%)
Psychiatric ward	26 (26%)
Other psychiatric services	12 (12%)
Police/judicial authorities	5 (5%)
Informal access	11 (11%)
DUP (weeks)^2^	9.3 ± 21.6
Diagnosis^3^	
Non affective psychosis	42 (42%)
Affective psychosis	21 (21%)
Psychosis NOS	33 (33%)
Other	5 (5%)
Medical comorbidity^4^	15 (17%)
Internal pathologies	7 (8%)
Metabolic diseases	3 (3%)
Traumatic diseases	2 (2%)
Other	3 (3%)
Total	109 (100%)

^1^8 missing

^2^64 missing

^3^8 missing

^4^23 missing

Clinical information was available for 8 centres. We found significant differences between centres in relation to PtC. As shown in Table 5, psychiatric wards were the main source of referral in Bologna (41%), Foggia (69%) and Pavia (60%), while in Bari, Modena and Novara patients were predominantly sent to CMHCs by the ER or other hospital wards (77%, 42% and 57% respectively). Informal referral was found almost exclusively in Bologna (18%) and Modena (21%). The most frequent diagnosis was non-affective psychosis in Bologna (50%), Modena (54%) and Pavia (80%), while it was affective psychosis (47%) in Bari and psychosis NOS (63%) in Foggia. Only 4 centres evaluated DUP: the average DUP in Novara was 1.8 weeks (± 0.8), while in Bologna it was 10.3 weeks (± 6.9) and in Modena 14 weeks (± 39.2).

**Table 5:** Clinical features of the sample by study centre

	BOL	CAG	CHV	BAR	FOG	MOD	NOV	PAV	*p*
Referral GP ER/General hospital Psychiatric ward Other psychiatric services Police/judicial authorities Informal access	4 (18%) 3 (14%) 9 (41%) 2 (9%) - 4 (18%)	1 (100%) - - - - -	1 (50%) 1 (50%) - - - -	- 13 (77%) 2 (12%) - 1 (6%) 1 (6%)	- 3 (19%) 11 (69%) 2 (13%) - -	1 (4%) 10 (42%) - 5 (21%) 3 (13%) 5 (21%)	- 8 (57%) 1 (7%) 3 (21%) 1 (7%) 1 (7%)	1 (20%) 1 (20%) 3 (60%) - - -	< 0.001
Diagnosis Non affective psychosis Affective psychosis Psychosis NOS Other	11 (50%) 5 (23%) 5 (23%) 1 (5%)	- - 1 (100%) -	1 (50%) 1 (50%) - -	5 (29%) 8 (47%) 4 (24%) -	2 (13%) 2 (13%) 10 (63%) 2 (13%)	13 (54%) 3 (13%) 6 (25%) 2 (8%)	6 (43%) 2 (14%) 6 (43%) -	4 (80%) - 1 (20%) -	0.098
Substance use^*^	7(33%)	-	-	3(30%)	2(13%)	4(18%)	4(29%)	1(25%)	0.842
Total	22 (22%)	1 (1%)	2 (2%)	17 (17%)	16 (16%)	24 (24%)	14 (14%)	5 (5%)	

*BOL* Bologna, *CAG* Cagliari, *CHV* Chivasso (TO), *BAR* Bari, *FOG* Foggia, *MOD* Modena, *NOV* Novara, *PAV* Pavia

^*^12 missing

## Discussion

### Socio-demographic Characteristics

East European migrants are the most numerous group of origin in our sample as well as in the general migrant population of Italy: migrants come to Italy mainly from European countries (both EU, 27.4% and non-EU, 23.4%), 22.1% from Africa, followed by Asian (18.8%) and American (8.3%) migrants [22]. The distribution of the migrants’ groups of origin in the samples collected by the 9 centres varies accordingly with the differences found in the general population of those centres. Despite different distributions, FEP migrants recruited by the PEP-Ita study have very similar socio-demographic characteristics in the 9 involved centres.

One interesting result of our study is the “high functioning portrait” of the migrants with FEP in Italy: they are generally highly educated, in their thirties, and are quite frequently employed and in a current or past stable affective relationship. Thus, migrants with FEP in Italy seem to have a higher personal and social functioning compared to Italian born natives with FEP, who are very frequently single, less educated, unemployed, and living with parental families [6, 7]. This result pushed us to generate the hypothesis that there is a higher burden of social- environmental risk factors at the psychosis onset in migrants compared to natives. Further study with a direct control group of natives and healthy people is needed to test this hypothesis.

As expected, FEP migrants compared with migrants in the general population are more often male (55% of our sample, 47% of migrants’ general population) and unemployed (34% vs 14.1%). Male gender and unemployment are 2 well known socio-demographic characteristic frequently found in FEP patients. We did not find other relevant differences between migrants in our sample and those in the general population. In particular our finding about education shows around half of the sample has high school or more, which is consistent with the level of education found in migrants in the general population (40.5% high school license; 9% university degree) [22].

Interestingly, the mean age of FEP migrants in our sample is similar to those of migrants in the general population (31.8 vs 31.1) and similar to the mean age found by previous studies on Italian natives with FEP [6] and by studies conducted in other countries [23, 24].

### Clinical Characteristics and PtC

Around 2/3 of FEP migrants were referred to CMHCs after emergency access to a general hospital or after psychiatric admission and only around 20% had a GP referral or direct access to mental health services. This result is consistent with a large body of evidence which indicates that migrants in western countries have different access to and low utilization of community mental health centers (CMHCs) despite the high prevalence of mental disorders [25–28].

The mean DUP of 9 weeks found in our sample is relatively short in comparison with other studies [29]. This result, along with the emergency-pattern of psychiatric services’ use and the high personal and social functioning found in our FEP migrants, could indicate an acute onset of psychosis in this population. However, data on DUP were missing for 4 centres, as its assessment was part of the optional levels of the PEP-Ita study.

Non-affective psychoses represented the largest diagnostic group in our sample, in accordance with previous evidence [6, 7]. The second most frequent diagnostic group was NOS-psychosis; in our opinion this second diagnostic cluster could reflect the discordant clinical presentation of these patients, characterized by psychotic clinical symptomatology and high personal and social functioning. Notably, none of the migrants included in our sample received a diagnosis of substance related psychosis. Moreover, a large proportion of FEP migrants presented to CMHCs with medical comorbidities.

Finally, our findings show a significant amount of heterogeneity across the nation regarding FEP migrants’ PtC. This is consistent with our previous study [10], where differences in the PtC between the various centres were not explained by the socio-demographic factors taken into account. One possible explanation for inter-centre variations in migrant PtC might be connected to the degree of awareness of “mental health” services and social services on the part of immigrants in each region, as well as to the degree of cultural competence in different CMHCs.

## Conclusions

Migration is a rapid and growing phenomenon in western countries and the association between psychotic disorders and migration history is a public health concern. In Italy, Mental Health Services are working to identify appropriate resources and organizational methods to respond to the mental health needs of migrants. The emergency-pattern of the CMHCs utilization, together with a mean duration of stay in Italy of around 9 years, the mean age at first CMHCs contact of around 31 years (very similar to the mean age of migrants in general population) and the high personal and social functioning found in migrants with FEP by our study allow us to hypothesize that they are a highly resistant population. It is probable that the onset of psychosis in migrants occurs only when the burden of negative psychosocial factors (such us racism, social isolation, discrepancy between expectations and achievement) overcomes a high threshold. In our study, FEP migrants frequently access the CMHCs with a medical comorbidity: this indicates the need to develop a more appropriate policy of health care delivery for the migrants population in Italy. Further studies with both native and healthy migrants control groups are needed to better understand psychotic disorders in migrants.
